# Optical attenuation coefficient decorrelation-based optical coherence tomography angiography for microvascular evaluation of Alzheimer’s disease on mice

**DOI:** 10.1117/1.NPh.12.1.015013

**Published:** 2025-03-12

**Authors:** Ben Xiang, Ning Ding, Huiwen Jiang, Jian Liu, Yao Yu, Jingmin Luan, Yuqian Zhao, Yi Wang, Yanqiu Yang, Cheng Ji, Fengwen Wang, Zhenhe Ma

**Affiliations:** a Northeastern University, College of Information Science and Engineering, Shenyang, China; b Northeastern University at Qinhuangdao, School of Control Engineering, Qinhuangdao, China; c Hebei Key Laboratory of Micro-Nano Precision Optical Sensing and Measurement Technology, Qinhuangdao, China; d Qinhuangdao Haigang Hospital, Qinhuangdao, China

**Keywords:** amyloid β, Alzheimer’s disease, optical coherence tomography, optical attenuation coefficient decorrelation, deep cortical microvasculature

## Abstract

**Significance:**

The deep cortical microvasculature is closely linked to the pathogenesis of Alzheimer’s disease (AD). However, tail artifacts from superficial cortical vessels often interfere with detecting deep vessels in optical coherence tomography angiography (OCTA) imaging. A more accurate method to assess deep cortical vasculature is crucial for understanding its relationship with AD onset.

**Aim:**

We aim to reduce superficial vessel artifacts in OCTA imaging and improve the visualization and analysis of deep cortical microvasculature in an AD mouse model.

**Approach:**

We introduced the optical attenuation coefficient decorrelation (OACD) method for OCTA, effectively reducing tail artifacts from superficial cortex vessels. This method was used to visualize and quantitatively analyze deep cortical microvasculature *in vivo* in a mouse model of AD.

**Results:**

The OACD method significantly reduced superficial vessel artifacts, leading to clearer imaging of the deep cortical vasculature. Quantitative analysis revealed that changes in the deep cortical microvasculature were more pronounced than in the superficial vasculature, suggesting a more direct involvement of the deep vessels in AD progression.

**Conclusions:**

The proposed OACD method enhances OCTA imaging by reducing tail artifacts from superficial vessels, facilitating improved assessment of deep cortical microvasculature. These findings suggest that deep cortical vascular changes may play a key role in the pathogenesis of AD, offering potential insights for early detection and monitoring of AD progression.

## 1 Introduction

Alzheimer’s disease (AD) is a chronic, progressive neurodegenerative condition that constitutes the predominant form of dementia, encompassing 60% to 80% of cases. AD manifests with an insidious onset; its evolution is nonreversible once initiated. The preclinical phase can span multiple decades;^1^ consequently, many patients are already in the intermediate to advanced stages at the point of clinical diagnosis. Presently, there remains an absence of established therapeutic strategies.^1^ The etiopathogenesis of AD is intricate, with multiple prevailing theories regarding its causative factors and fundamental mechanisms, yet a consensus remains elusive. In recent two decades, the amyloid-β (Aβ) toxicity paradigm has garnered widespread endorsement within the scientific realm. Intriguingly, a mere few years before identifying Aβ protein variants, certain researchers posited that cerebral microangiopathy and inflammatory processes might be intrinsic initiators of AD.^2^^–^^5^ Within the pathophysiological framework of AD, deep cerebral microvasculature assumes an incrementally critical function. The microvasculature not only orchestrates the provision of essential nutrients and oxygen to cerebral tissues but also oversees the evacuation of metabolic waste products. Anomalies in vascular structures, particularly in cerebral regions, are postulated to have a direct nexus with AD pathogenesis.^6^ In our previous study, we reported the direct effects of cerebrospinal fluid-Aβ on cerebrovascular morphology and brain tissue.^7^ Due to the technique limitation, microvasculature change of the deep cortex was not evaluated specifically.

*In vivo* imaging of small animal brains has greatly propelled the rapid advancement of neuroscience. Some nonoptical vascular imaging techniques, such as magnetic resonance imaging and computed tomography, offer lower resolution, rendering them unsuitable for microvascular imaging.^8^^,^^9^ Optical vascular imaging techniques, such as fluorescein angiography, are invasive, requiring the injection of a fluorescein contrast agent.^10^ Speckle imaging offers shallow imaging depth and is ineffective for imaging deeper vasculature.^11^ By contrast, optical coherence tomography (OCT) is a noninvasive optical imaging technique that requires no contrast agent. Furthermore, OCT boasts high imaging resolution, a broad imaging range, and sufficient penetration depth. It stands out as the optimal choice for monitoring tissue characteristics throughout the mouse brain.^12^^–^^14^ Optical coherence tomography angiography (OCTA) is a functional extension of OCT, providing 3D maps of blood perfusion in tissues down to the capillary level.^15^ OCTA has been successfully employed for imaging microvascular networks in various *in vivo* tissues, such as the brain^14^ and retina.^16^

Although OCTA has seen rapid adoption across various applications, it still confronts challenges. Specifically, the vasculature of upper tissue layers leads to tail artifacts in flow signal extraction, obscuring the detection of deeper microvasculature and affecting vascular quantification.^17^^–^^19^ Methods have been proposed to mitigate the effects of this tail artifact. Jia et al.^20^ introduced a slab-subtraction approach. This method first acquires a binary mask of large vessels in the superficial layer and then subtracts the signal at the positions of large vessels from the deep layer. This approach effectively eliminates the tail artifacts of large vessels but may not completely remove the tail artifacts for some small vessels. Choi et al.^21^ proposed a mean subtraction method, which suppresses the signal in the area below large vessels using a weighted average of all pixels in each A-line. However, this method may compromise the information of deep blood flow. Wang et al.^22^ proposed a sacPR-OCTA method that eliminates tail artifacts through signal attenuation compensation. However, the relationship among signal attenuation, reflectance, and optical distance is complicated and unsuitable for structurally complex cerebral vessels. Baran et al.^23^ noticed that the OCT structural signal below vessels is typically weaker, whereas the OCTA signal below vessels is stronger. Therefore, they created an adaptive mask using deep-layer OCT en-face structural images to suppress the tail artifacts of vessels. Although it has produced good results, this method may suppress signals in capillaries that are not beneath large blood vessels, affecting the detection of deep microvessels. These methods perform well in eliminating tail artifacts in large vessels and are useful for quantifying fundus blood vessels. In studies on AD, we focus more on deep cerebral cortex microvasculature. The surface of the cortex has abundant vessels, most of which are medium and small. The tail artifacts from these medium and small vessels also affect the deeper vascular quantification and appear as background noise in OCTA images. Furthermore, the power fluctuation of the light source introduces background noise as OCTA eventually extracts flow signals by different calculations between B-scan images. Reducing this background noise can improve the accuracy of vascular quantification in the deeper cortex, hence benefiting studies on cerebral microvascular modifications of AD.

The optical attenuation coefficient (OAC) is a pivotal biophysical parameter. Inherently, the OAC represents the degree of light attenuation as it traverses through biological tissue, thus serving as an intrinsic marker of tissue characteristics. By assessing the OAC, distinctions can be made between various tissue types affected by pathological conditions, facilitating the detection and quantification of a spectrum of diseases,^24^ such as the imaging of atherosclerotic plaques^25^ and the evaluation of glaucoma.^26^ Among the techniques developed to compute the OAC, the depth-resolved (DR) method proposed by Vermeer et al.^27^ is predominantly employed. Addressing diverse challenges and requirements, other methods, such as depth-resolved confocal,^28^ optimized depth-resolved estimation (ODRE),^29^ and overestimation-free depth-resolved attenuation estimation,^30^ have been introduced. Notably, although the OAC is typically harnessed to differentiate tissues influenced by disease,^31^^–^^34^ its application in vascular imaging remains uncharted. Given that OAC reflects an intrinsic property of tissues unaffected by incident light and adjacent tissue perturbations, it possesses the potential to mitigate the tail artifact phenomenon in OCTA.

This study proposed an OAC-based decorrelation approach, aiming to suppress vascular tail artifacts and thereby extract minutiae of capillaries concealed within such effects. The present method is employed to visualize deep cortex capillaries of murine models *in vivo* using swept-source OCT (SS-OCT). The primary purpose of this study centers on exploiting OAC to extract and monitor deep cortex microvasculature in early AD stages. Metrics including vascular perfusion density (VPD), vascular length (VL), vascular average diameter (VAD), vascular tortuosity (VT), number of branching nodes, and number of endpoints were assessed. Discrepancies in vasculature between the superficial and deep cortex were compared, and the morphological changes of vasculature in the deep cortex were more notable.

## 2 Methods

### 2.1 Experimental System

In this study, a wide-range SS-OCT system with a central wavelength of 1310 nm was used to visualize the vasculature of the mouse cortex, which has been introduced in our previous publications.^35^ Briefly, the swept rate of the light source is 200 kHz, and the spectral bandwidth is 100 nm, providing an axial resolution of 7.5  μm in air. Each cross-sectional B-scan image consists of 1000 A-lines. At each position (Y direction), 10 B-scans were captured repeatedly for flow signal extraction. The scan was performed at 800 positions to achieve 3D-OCTA imaging. The imaging area covered the entire mouse cerebral ($∼12(X)\times 10(Y)\;{\mathrm{mm}}^{2}$).

### 2.2 OCT and OCTA Imaging

OCT is based on low-coherence interference. The interference spectrum between the reference beam and backscattered light from the sample can be expressed as

$$I(k)=S(k)(E_{R}^{2}+2E_{R}[\int_{-\infty }^{\infty }a(z)\cos(2kz)dz]+\int_{-\infty }^{\infty }\int_{-\infty }^{\infty }a(z)a(z^{\prime })\exp[i2k(z-z^{\prime })]dz\;dz^{\prime }),$$ (1)

$$z=\int_{0}^{d}n(d^{\prime })dd^{\prime },$$ (2)

where S(k) is the spectrum of the light source, $E_{R}$ is the amplitude of the reference beam, k is the wavenumber, d is the distance relative to the reference mirror, z is the optical path difference, a(z) is the amplitude of the backscattered light at z, and n(d) is the refractive index distribution of the sample. In Eq. (1), $E_{R}^{2}$ represents the auto-correlation component of the reference beam (DC term), whereas $2E_{R}[\int_{-\infty }^{\infty }a(z)\cos(2kz)dz]$ is the interference term which encoded sample information a(z). By performing a fast Fourier transform in Eq. (1) and neglecting the DC term, we obtain

$$F(I(k))=F(S(k))⊗(2E_{R}(a(z)+a(-z))),$$ (3)

where F(I(k)) is a complex signal corresponding to the sample’s backscattering distribution. The amplitude of F(I(k)) is used to construct the OCT structure image.

OCTA is based on motion contrast imaging, i.e., motions of red blood cells (RBCs) are extracted from different B-scans. At each position, multiple B-scans are acquired repeatedly. The difference between these images is obtained by direct subtraction or decorrelation, corresponding to the blood flow. The operation is performed at multiple positions for 3D OCTA rendering.

### 2.3 Mechanism of Tail Artifacts in OCTA

In OCT, the probe light propagates through tissue. The tissue absorbs and scatters the light simultaneously, resulting in light attenuation with depth increase. The backscattered light (a portion of the scattered light) at different depths a(z) is used for OCT imaging. Based on the Beer–Lambert–Bouguer Law, a(z) is expressed as

$$a(z_{j})=a_{0}\cdot R_{Z_{j}}\cdot e^{-2\overset{j-1}{\underset{i=1}{\sum }}\mu_{i}z_{i}},$$ (4)

where $a_{0}$ is the incident light intensity, $R_{Z_{j}}$ is the reflectivity of the tissue at layer $z_{j}$, which is determined by the refractive index difference between adjacent layers, and $\mu_{i}$ is the attenuation coefficient of the tissue. In Eq. (4), $a_{0}\cdot e^{-2\overset{j-1}{\underset{i=1}{\sum }}\mu_{i}z_{i}}$ represents the light intensity incident at the optical path difference $z_{j}$. According to Eq. (4), it is evident that the intensity of incident light at each tissue layer is influenced by the attenuation of overlying tissue layers, which in turn affects the backscattered light $a(z_{j})$. In the OCT structure image, blood flow causes $a(z_{j})$ change among repeated B-scans. OCTA calculates differences to preserve the dynamic blood flow signals while suppressing the static signals from stationary tissue, thus realizing vascular imaging.

**Fig. 1:**
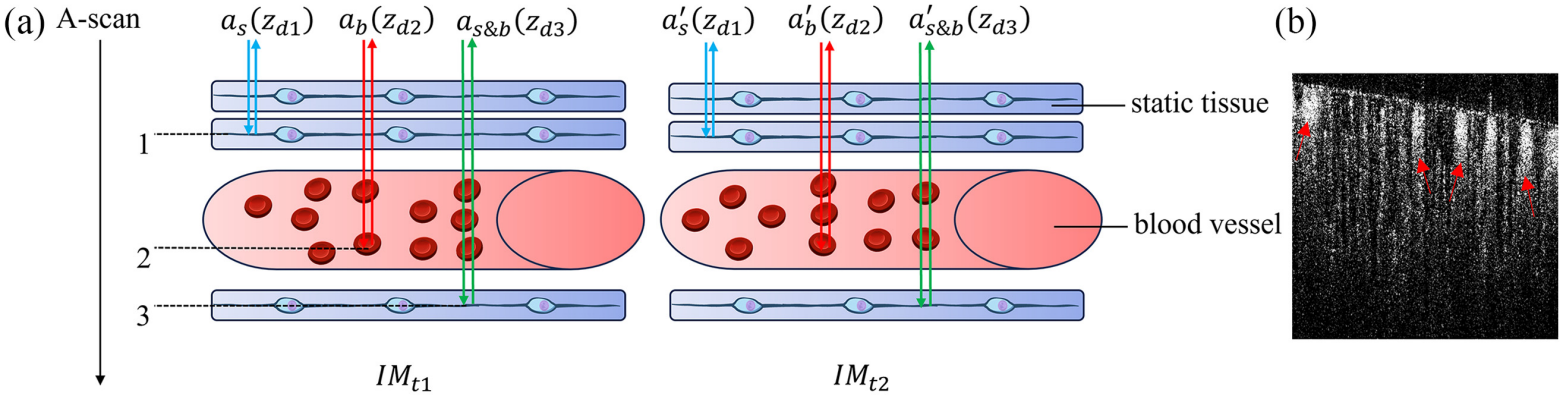
Tail artifacts generation in OCTA. (a) Two B-scans acquired at the same position and (b) tail artifacts in the B-scan flow signal extraction (red arrows).

Figure 1 illustrates the mechanism of OCTA and the generation of the tail artifacts in OCTA. Figure 1(a) shows the schematic diagrams of two B-scan images (${\mathrm{IM}}_{t1}$ and ${\mathrm{IM}}_{t2}$) acquired at the same position. In ${\mathrm{IM}}_{t1}$ and ${\mathrm{IM}}_{t2}$, vessels are embedded in the static tissue. The blue arrows in the two images represent light backscattered from the static tissue above the vessel, the red arrows represent light backscattered from the flowing RBCs, and the green arrows represent light backscattered from the static tissue beneath the vessel. For the condition of blue arrows, the intensity of backscattering light $a_{s}(z_{d1})$ does not change between the two B-scan images because there is no tissue motion in the propagation path of the blue arrows. Thus, this static tissue can be eliminated by the OCTA algorithm. For the condition of red arrows, the incident light on RBCs does not change between the two B-scan images, whereas the motion of RBCs causes the reflectivity ($R_{Z_{j}}$) to change [see Eq. (4)]. Eventually, the intensity of backscattering light from the flowing blood changes between the two B-scans. The OCTA algorithm extracts the amplitude change to visualize the blood vessels. For the condition of green arrows, the reflectivity [$R_{Z_{j}}$ in Eq. (4)] does not change between the two images because the light is backscattered from the static tissue. However, the intensity of incident light changes because of the blood flow, i.e., the incident light passes through different blood RBCs. Thus, the intensity of backscatter light from static tissue beneath the vessel also changes. After OCTA calculation, the change is extracted and exhibited as a tail artifact. Though the previous analysis is based on the light amplitude, the flow of RBCs within vessels can also induce a phase change in the OCT signal, suggesting that phase-based OCTA exhibits tail artifacts similarly.

### 2.4 Calculation of OAC and OAC Decorrelation

The tail artifact arises from the influence of the upper blood flow on the underlying tissue during light propagation. If a tissue intrinsic parameter (irrelevant to light propagation) is used to extract blood flow, we can effectively suppress tail artifacts. OAC is an intrinsic property of the tissue, independent of incident light intensity. In this study, OAC takes the place of amplitude (or phase) on blood flow extraction to mitigate the tail effect in OCTA.

It is crucial to consider the confocal characteristics of OCT systems before calculating the OAC.^36^^,^^37^ It will affect the depth distribution of the light intensity signal with depth variation, thus affecting the accuracy of attenuation measurement. In our study, we employed the confocal axial point spread function (PSF) proposed by Leeuwen et al.^38^ to eliminate the influence of confocal characteristics, as described below:

$$h(z)={\left({\left(\frac{z-z_{cf}}{z_{R}}\right)}^{2}+1\right)}^{-1},$$ (5)

$$z_{R}=\alpha \pi nw_{0}^{2}/\lambda ,$$ (6)

where z is the signal depth, and the function h(z) is the axial PSF, $z_{\mathrm{cf}}$ is the position of the confocal gate, which was recorded during the experiment. $z_{R}$ is the “apparent” Rayleigh length used to characterize the axial PSF. $w_{0}$ is the minimum beam radius, λ is the center wavelength of the light source, and α is used to distinguish specular reflection (α=1) from diffuse reflection (α=2). The influence of confocal characteristics could be removed by dividing the intensity of OCT signals by axial PSF h(z), improving the accuracy of OAC estimation, particularly for depth-resolved methods.

There are two primary methods for OAC calculation: curve fitting and depth-resolution estimation. Curve fitting is accurate for homogeneous samples but may compromise depth resolution, making it unsuitable for vascular imaging. To address the limitation of depth resolution, Vermeer et al.^27^ introduced a depth-resolved OAC estimation method, which is described as

$$\mu [z]=\frac{I[z]}{2\Delta \sum_{i=z+1}^{N}I[i]},$$ (7)

where μ[z] (expressed in ${\mathrm{mm}}^{-1}$) is the OAC value, I[z] is the OCT signal intensity of a pixel at z, Δ is the pixel size, and N is pixel numbers within a certain depth range. The factor of 2 is due to the light propagating through the tissue twice. The estimation is accurate in the areas with high-intensity signals (e.g., superficial layers). With the depth increase, the estimation will produce errors because of the intensity attenuation or finite number of pixels in Eq. (7). To minimize computational errors in OAC, we proposed the ODRE method previously.^39^ Each pixel in the OCT dataset was converted to the corresponding OAC value using the equation

$$\mu [z]=\frac{I[z]}{2\Delta \sum_{i=z+1}^{N}I[i]+\frac{I[N]}{\mu [N]}},$$ (8)

where I[N] is the OCT signal of the last point N and μ[N] is the corresponding OAC. To determine μ[N], the data taken from the end of the imaging depth were fitted to an exponential curve with a fitting model of y=a·exp(−2μz)+b. Considering that the noise floor can cause inaccurate fitting μ[N], we increase the fitting accuracy by removing low-noise floor areas.^30^ The steps are: (1) average multiple adjacent A-line signals to improve the signal-to-noise ratio; (2) perform statistical analysis on the end part of the signal and use the average fluctuation of the tail signal strength as the benchmark for the noise floor intensity; (3) find the boundary along the depth direction where the signal strength is 5 dB higher than the noise floor and define this position as the effective range of the signal; (4) exclude the depth range dominated by the noise floor and only retain the signal portion significantly higher than the noise for subsequent attenuation coefficient calculations. By clarifying the boundary point between noise and signal, the interference of low signal-to-noise ratio areas on the calculation is eliminated, making the fitting of μ[N] more accurate and thus making the calculation of OAC more accurate. A more precise OAC can better suppress tail artifacts. In Eq. (8), ODRE calculates the ratio of the OCT signal value I[z] at depth z to the cumulative sum of all OCT signal values below that depth, and the calculated OAC is not affected by signals above depth z. Thus, OAC is a potential parameter for tail artifact suppression in OCTA imaging. The OAC decorrelation (OACD) is performed among B-scans acquired at the same position to extract the flow signal

$$\overset{¯}{D}(x,z)=1-\frac{1}{M-1}\overset{M-1}{\underset{m=1}{\sum }}\frac{{\mathrm{OAC}}_{m}(x,z){\mathrm{OAC}}_{m+1}(x,z)}{\left[\frac{1}{2}{\mathrm{OAC}}_{m}(x,z{)}^{2}+\frac{1}{2}{\mathrm{OAC}}_{m+1}(x,z{)}^{2}\right]},$$ (9)

where x and z are lateral and depth indices of the B-scan images, m denotes the B-scan slice index, and M is the number of repeated B-scans at each position. OAC is an intrinsic tissue parameter and independent of the incident light intensity. Thus, another advantage of OACD is that it can effectively suppress the background noise of OCTA introduced by the instability of light sources.

### 2.5 Deep Cortex Vascular Visualization

**Fig. 2:**
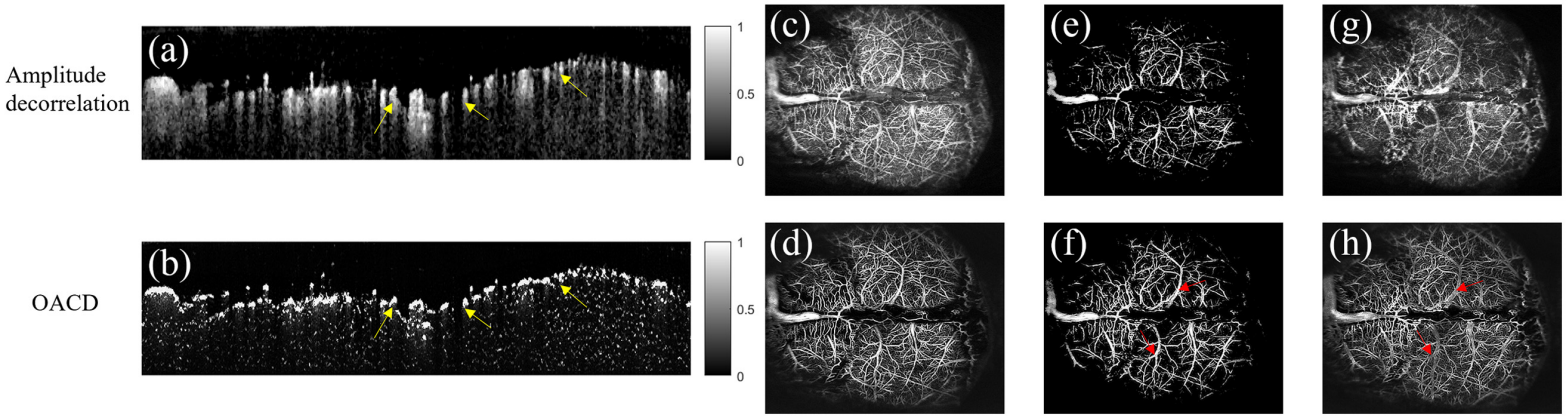
Comparison of wide-field OCTA images between amplitude decorrelation and OACD. (a), (c), (e), (g) Amplitude decorrelation results. (b), (d), (f), (h) OACD results. (a), (b) Cross-sectional tomograms of the mouse cerebral vasculature. (c), (d) Whole cortex vasculature. (e), (f) Superficial cortex vasculature. (g), (h) Deep cortex vasculature.

To evaluate the performance of the proposed OACD method, the mouse cortex was scanned for OCTA imaging. Figure 2 presents wide-field ($12(X)\times 10(Y)\;{\mathrm{mm}}^{2}$) OCTA images calculated by amplitude-decorrelation and OACD. The cortical thickness in mice is ∼700 to 900  μm. According to the depth, we divided the blood vessels of the mouse cortex into two groups: superficial and deep cortex vessels. The thickness of each vessel type is ∼450  μm. Figures 2(a), 2(c), 2(e), and 2(g) show the cross-sectional image and en-face image obtained by amplitude-decorrelation, whereas Figs. 2(b), 2(d), 2(f), and 2(h) show the cross-sectional image and en-face image obtained by OACD. Figures 2(a) and 2(b) show cross-sectional tomograms of the mouse cerebral vasculature. Compared with Figs. 2(a) and 2(b), we can see that OACD significantly suppresses tail artifacts [yellow arrows in Figs. 2(a) and 2(b)]. Figures 2(c) and 2(d) are whole cortex vascular images, including both superficial and deep vessels. As shown in Fig. 2(d), OACD reduces background noise, significantly improving image quality. Figures 2(e) and 2(f) show superficial vascular images, whereas Figs. 2(g) and 2(h) depict deep vascular images. Compared with Figs. 2(g) and 2(h), OACD suppresses artifacts from larger vessels, allowing for the visualization of smaller vessels buried within the tail effects and providing the potential for accurate vascular quantification. However, despite significant suppression, some artifacts of large vessels cannot be eliminated [red arrows in Figs. 2(f) and 2(h)]. Additional processes were performed to better evaluate vasculature in the deep cortex, including extraction of large vessels from the superficial vessel image, pattern matching between the extracted big vessel and deep cortex vessel image to determine the tail artifact and subtraction of tail artifact from deep cortex vessel image. To quantitatively analyze the degree of tail artifact removal, we use vascular similarity as a measure of tail artifact removal.^22^ We measured vascular similarity using Pearson’s correlation coefficient (r).^40^ An r value close to 0 indicates better performance in removing tail artifacts. In addition, to evaluate image quality, values of peak signal-to-noise ratio (PSNR),^41^ structural similarity (SSIM),^42^ and gradient magnitude similarity deviation (GMSD)^43^ were computed quantitatively and compared between amplitude decorrelation and OACD. The quantitative results are shown in Table 1. We can see that the proposed OACD method performs better in tail artifact suppression than OCT amplitude decorrelation. At the same time, the OCTA image quality has also been improved, including enhancement of PSNR and SSIM values and reduction of the GMSD value.

**Table 1:** Results of image quality evaluation in different methods (means ± Std).

Parameters	Amplitude decorrelation	OACD
Pearson’s correlation coefficient	0.73 ± 0.07	0.18 ± 0.05
PSNR	15.43 ± 0.65	27.83 ± 0.62
SSIM	0.72 ± 0.05	0.85 ± 0.02
GMSD	0.14 ± 0. 01	0.10 ± 0.01

### 2.6 Parameter Quantification

**Fig. 3:**
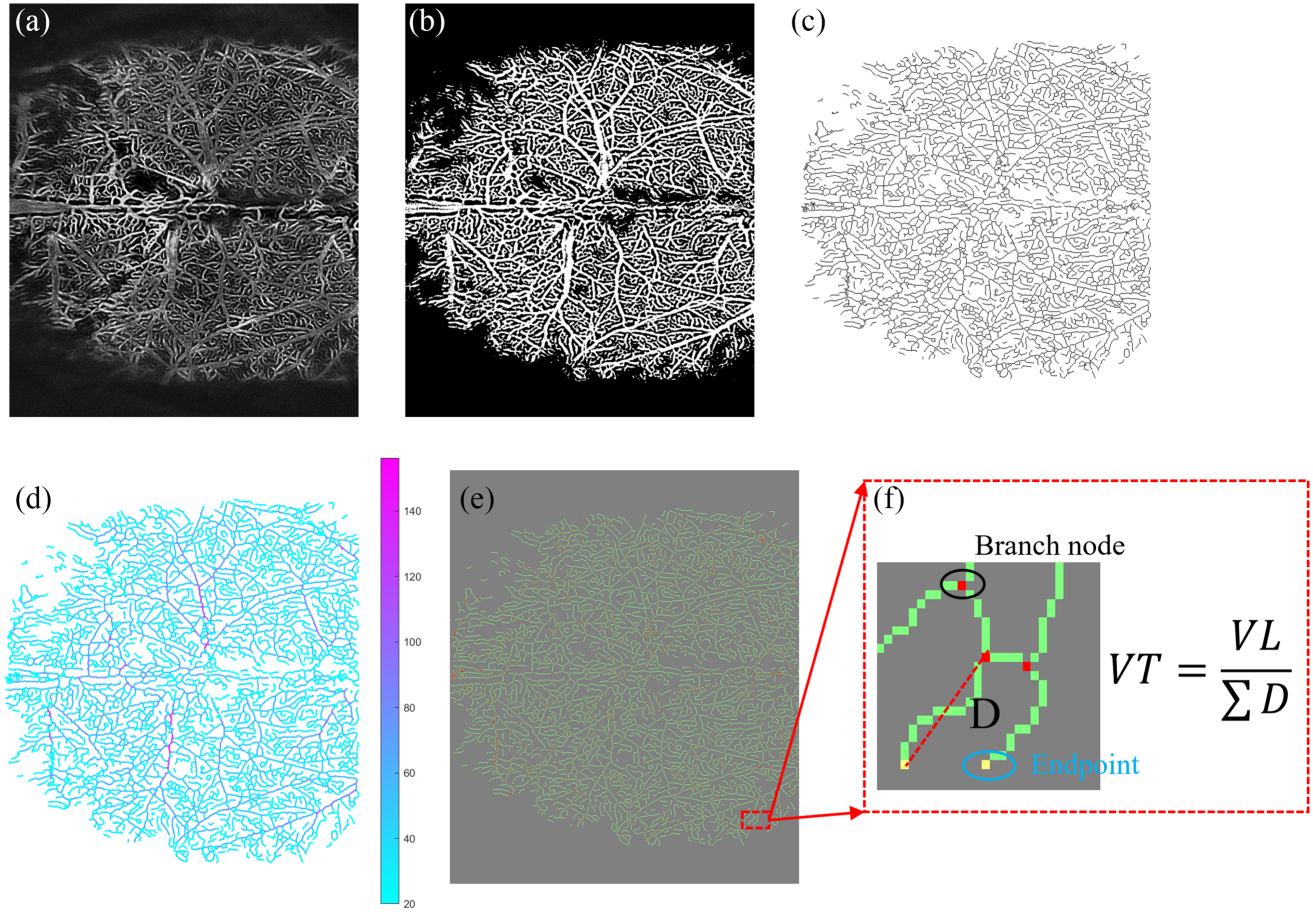
Image processing for vascular parameters quantification. (a) OCTA images. (b) Binarization result of panel (a). (c) Skeleton image of panel (b). (d) The skeleton image labeled with vessel diameters after distance transformation. (e) Vascular skeleton image with labeled vessel branches and terminals (red dots: branch nodes, yellow dots: endpoints). (f) Enlarged view of the red box in panel (e).

Four morphological parameters were selected to assess vascular status: VPD, VL, VAD, and VT. The “Local Adaptive Region Growth” algorithm^7^ is applied to the en-face OCTA image [Fig. 3(a)] for vascular segmentation, resulting in the binarized vascular image [Fig. 3(b)]. The advantage of this algorithm is that it can adaptively adjust the discrimination criteria based on the characteristics of the target and background pixels in the local area. This dynamic adjustment enhances segmentation accuracy by avoiding the erroneous classification of background as blood vessels in cases where both background and vessel signal intensities are elevated while also successfully detecting weak vessel signals in scenarios where both intensities are low. Then, we extract the vascular skeleton and distance transformation from the binary vascular image using a morphology-based refinement algorithm. The skeleton extraction process includes the following steps: (a) Identify boundary pixels in an image that exhibit specific structural features. (b) Iteratively remove these boundary pixels while maintaining the connectivity of the structure, gradually thinning the structure. (c) The final output is a skeleton representation of the input structure, with key features retained as lines with one-pixel width. We obtained vascular skeleton images [Fig. 3(c)]. To simultaneously reflect the diameter of blood vessels, we applied distance transformation to the binarized vascular images and obtained a vascular skeleton image with diameter information [Fig. 3(d)]. To compute VT, all endpoints and branch points were detected and classified from the vascular skeleton image [Fig. 3(e)]. Figure 3(f) shows the enlarged area (dashed red box) in Fig. 3(e), where branch points are yellow and endpoints are blue. Subsequently, VPD, VL, VAD, and VT were calculated. VPD is defined as the ratio of the vascular area to the total area in the binarized image and serves as a parameter for assessing blood flow perfusion. VL represents the total length of the vascular skeleton. VAD is obtained by dividing VL by the sum of vessel diameters. VAD is a parameter to gauge vessel diameter and exhibits sensitivity to vascular dilation. It is a powerful tool for discerning localized vessel dilation and offering diagnostic insights for vascular abnormalities. VT is derived by dividing VL by the sum of the Euclidean distances (linear lengths) between branch points and endpoints.^44^^,^^45^ VT is a pivotal parameter reflecting vessel curvature: a straight vessel possesses a tortuosity value of 1, and as a vessel becomes more tortuous, its value increases.

### 2.7 Animal Preparation

All procedures were approved by the Institutional Animal Care Committee of Northeastern University. All efforts were made to minimize animal suffering and to reduce the number of animals used. C57Bl/6NCr male mice (N=12) between 8 and 12 weeks of age were used in all experiments. Animals were randomized into two groups: the control and AD groups. Mice were anesthetized using sodium pentobarbital (3%, 5  mg/100  g, IP). The anesthetized mouse was secured on the ST-5ND-C stereotaxic platform using ear bars and a holder. After trimming the fur, an incision was made down the cranial midline, revealing the interparietal section of the skull. The skull was ground to a 0.1- to 0.2-mm thick to get clear OCT images. $A\beta_{1-42}$ (A9810; Sigma-Aldrich, St. Louis, Missouri, United States) was pre-dissolved in 10% DMSO/PBS and overnight incubated at 37°C. This peptide solution (1  μg/μL) was then administered into the mouse’s lateral ventricle at a consistent pace of 1  μL/min. Based on The Mouse Brain in Stereotaxic Coordinates (2001), the injection’s stereotactic coordinates are set at 0.5-mm posterior, 1-mm lateral, and 2.5-mm beneath the bregma.^46^^,^^47^ Controls consisted of mice age- and sex-matched to AD mice, subjected to skull grinding and injection of an equal volume and concentration of DMSO/PBS at the same location.

### 2.8 Data Analysis and Statistics

Statistical analyses were conducted using GraphPad Prism (Graph-Pad Software Inc., San Diego, California, United States). All experiments were performed 12 times (unless otherwise specified), and data were presented as mean ± S.E.M. Statistical differences were evaluated using an unpaired two-tailed Student’s t-test when comparing only two groups. One-way analysis of variance was used to analyze within-group differences, with post-hoc comparisons conducted using the Newman–Keuls test. Values of p<0.05 were considered statistically significant. We monitored the superficial vasculature and deep microvessels to assess microvascular changes during AD progression. The vascular parameters VPD, VL, VAD, VT, number of branching nodes, and endpoints were compared for both groups.

## 3 Results

### 3.1 Deep Whole Brain Microvascular Imaging by SS-OCT

**Fig. 4:**
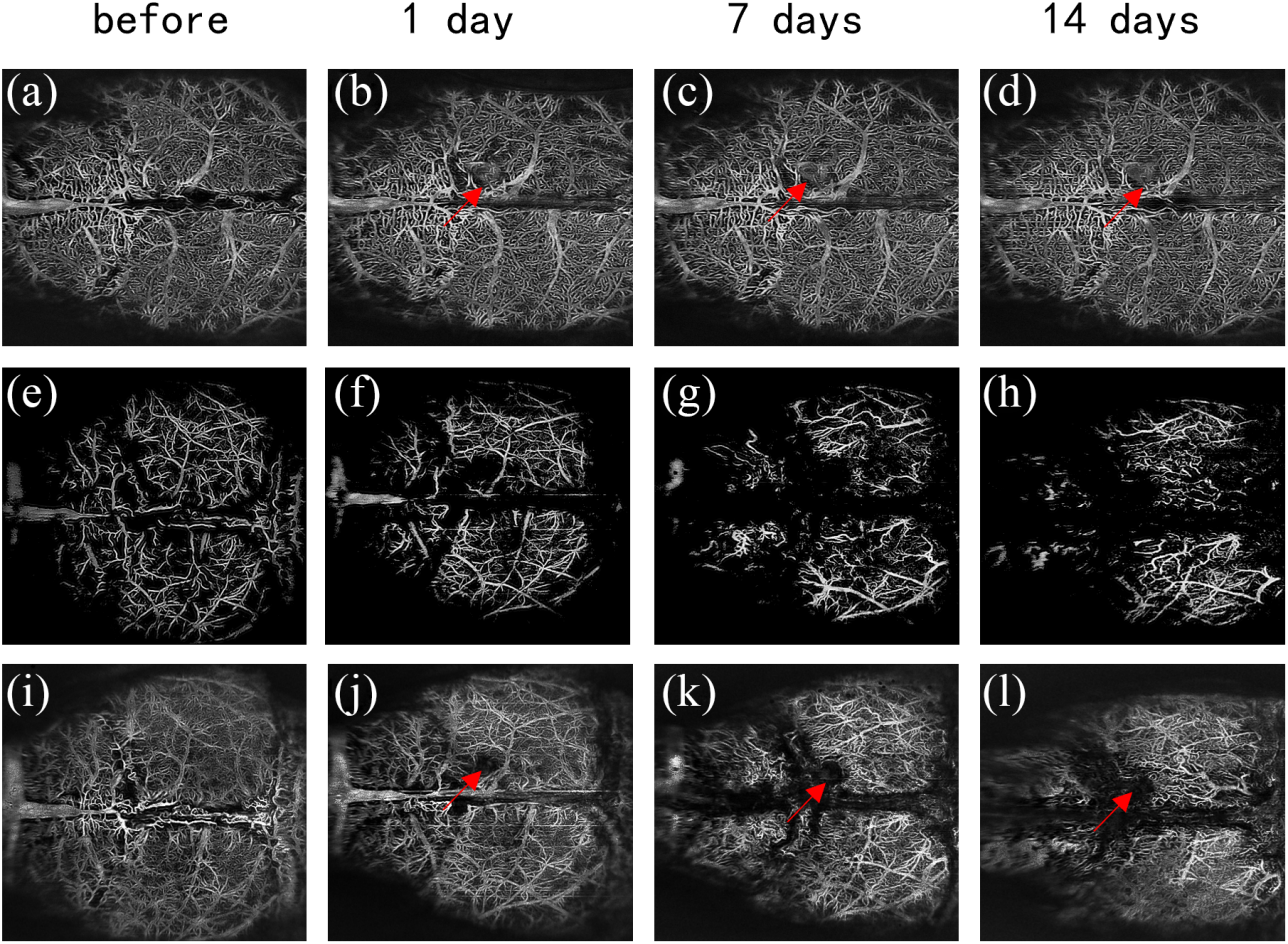
OCTA images at different time points after the ventricular injection. (a)–(d) Control group of deep cortex microvasculature. (e)–(h) Superficial cortex vasculature after Aβ injection. (i)–(l) Deep cortex microvasculature after Aβ injection.

Mice received Aβ peptide via ventricular administration, and continuous observation of the entire brain was carried out using SS-OCT for two weeks. To understand alterations in brain vascular structures, OCTA images were processed to illustrate binarized angiographies, VPD maps, and vascular skeleton representations, as depicted in Fig. 3. Figure 4 shows the OCTA images at different time points after the ventricular injection, i.e., Figs. 4(a)–4(d) for the deep cortex microvasculature of the control group, Figs. 4(e)–4(h) for superficial cortex vasculature after Aβ injection, and Figs. 4(i)–4(l) for deep cortex microvasculature after Aβ injection. As shown in Figs. 4(e)–4(h) and 4(i)–4(l), following peptide administration, a reduction in capillary density became evident and progressively intensified over time.^48^ However, there is almost no change in deep microvasculature in the control group. In the control group, we can see a small area of vessel absence around the injection spot [Figs. 4(a)–4(d) red arrows], which is due to physical trauma caused by injection. For the AD group [Figs. 4(i)–4(l)], blood vessel absence is distinct around the injection spot, and the vessel blank area increases over time (red arrows). The relatively high concentration of peptide around the injection site caused the destruction of cortical vessels, leading to their disappearance in the OCTA images.

### 3.2 Quantification of Cortical Vascular Morphology

**Fig. 5:**
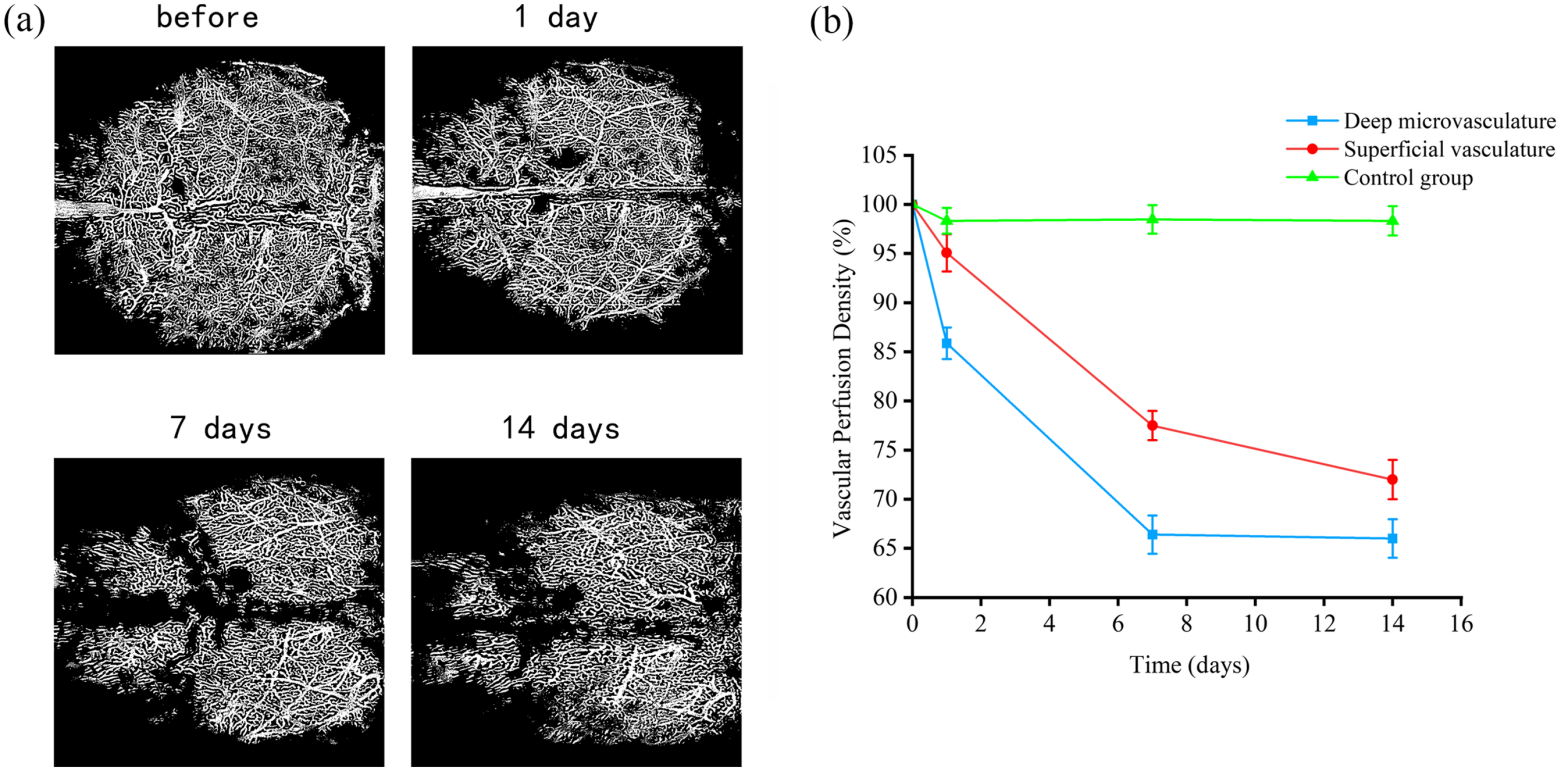
Progression of VPD as a function of time after amyloid peptide perfusion. Panel (a) presents the corresponding binarized angiograms at different time points. Data were expressed as percentage values relative to before treatment. Panel (b) shows the VPD % changes over time, where the red line represents the VPD changes in the superficial vasculature, the blue line indicates the VPD changes in the deep microvasculature, and the green indicates the VPD changes in the deep microvasculature in the control group.

Figure 5(a) presents binarized images of representative deep cerebral microvasculature in mice at 1, 7, and 14 days post-injection of amyloid-beta peptide. Quantitative analysis of the VPD was performed based on these binarized images. Both the binarized images [Fig. 5(a)] and the quantitative results [Fig. 5(b)] indicate a significant reduction in VPD following Aβ administration. On days 1, 7, and 14 after Aβ injection, the VPD for deep microvessels dropped to 85.9%, 66.4%, and 66% of baseline, respectively [Fig. 5(b), blue line]. Meanwhile, the VPD for the superficial vasculature decreased to 95.1%, 77.5%, and 72% of baseline, respectively [Fig. 5(b), red line]. VPD in the control group of deep microvessels remained mainly unchanged, with a decrease in VPD attributed to vascular injury incurred during injection [Fig. 5(b), green line]. The study revealed that the VPD in the deep microvessels declined more rapidly than in the superficial vasculature. These findings suggest that the deep microvasculature might exhibit heightened sensitivity to Aβ.

**Fig. 6:**
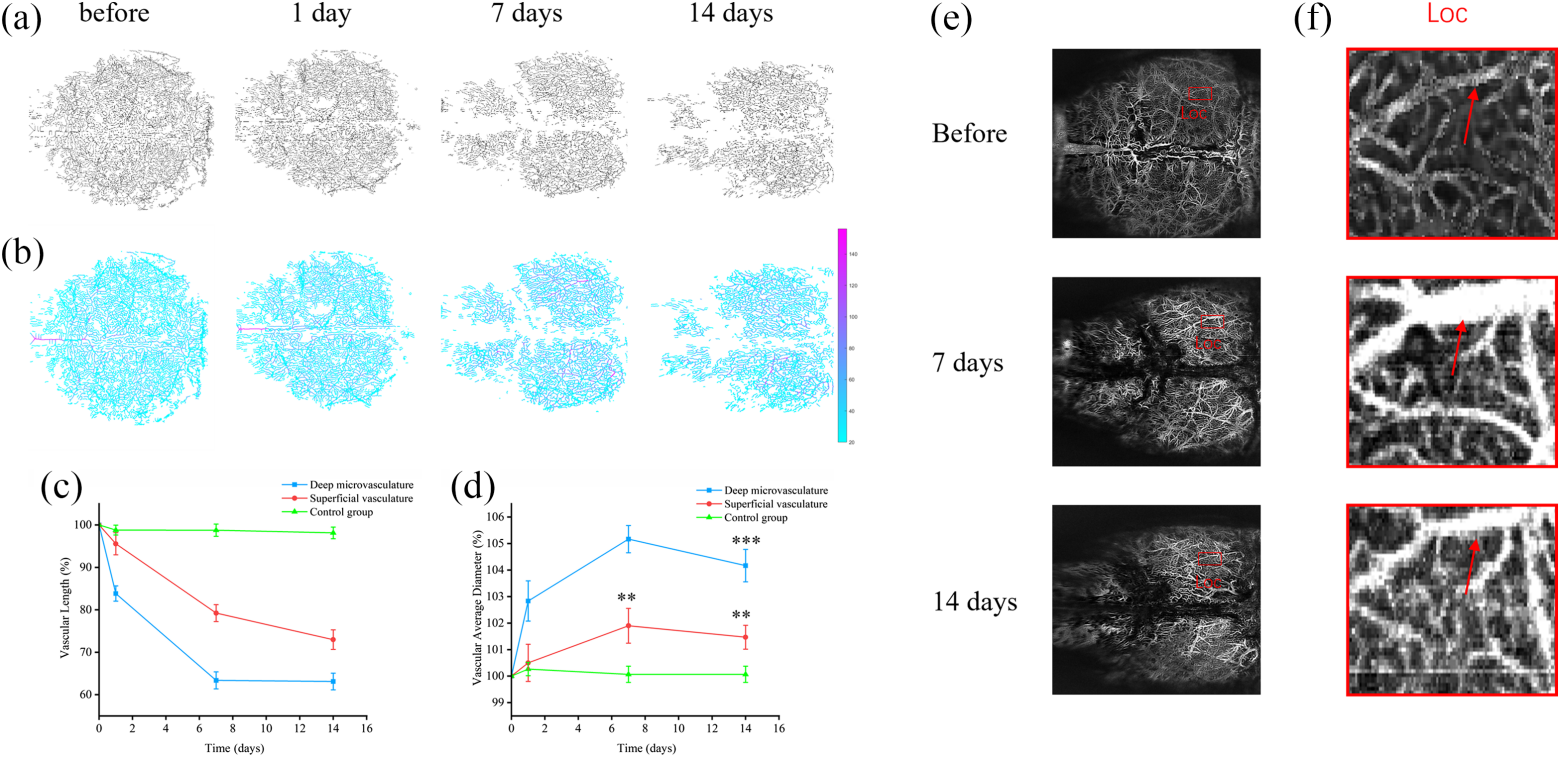
VL and VAD change after peptide injection. Panel (a) displays vascular skeleton images. Panel (b) displays a skeletonized image with vessel diameters annotated following distance transformation. Each pixel value corresponds to the vessel diameter. Panels (c) and (d) respectively show the VL% and VAD% change over time of the superficial vasculature (red lines), deep microvasculature (blue lines), and deep microvasculature in the control group (green line). Each bar represents the group mean ± S.E.M, significant differences between certain groups, and before denoted **p<0.01. (e) Areas where the vascular diameters have noticeably expanded compared with pre-injection measurements in the brain, indicated by red boxes. (f) Enlarged view of the red box in panel (e).

Next, we further explored the parameters VL and VAD, which could be associated with the observed decline in VPD. Vascular images underwent skeletonization and distance transformation, as illustrated in Figs. 6(a) and 6(b). VL and VAD were determined based on the vascular skeleton and binarized images. The results revealed that after Aβ administration, the VL of microvessels sharply decreased to 63.1% of its pre-injection value by day 14 [Fig. 6(c), blue line]. This decrease is more pronounced than the VL of superficial vasculature, which dropped to 73% on day 14 post-injection [Fig. 6(c), red line]. In particular, the deep microvessels exhibited a more rapid decline, especially between days 0 and 7 post-injection. In the context of VAD, we noted that some vessels exhibited dilation on day 7 post-injection and re-contracted by day 14 [red arrows in Fig. 6(f)]. However, compared with pre-injection levels, these vessels remained dilated, which may have contributed to a slight increase in VAD. Figure 6(d) presents the variations in VAD. The VAD of deep microvessels showed a slight increase to 105.2% of its pre-injection level on day 7 post-injection but decreased to 104.2% from day 7 to 14, demonstrating a significant difference compared with its pre-injection value (p=0.0003) [Fig. 6(d), blue line]. The superficial vasculature’s VAD displayed a slight but significant increase trend post-injection, reaching 101.5% of its pre-injection value by day 14 (p=0.0049) [Fig. 6(d), red line]. The VAD variations suggest that vascular dilation might predominantly occur in the deep microvessels. Compared with the AD group, there is no significant change in VL and VAD of deep microvasculature in the control group [green lines in Figs. 6(c) and 6(d)].

**Fig. 7:**
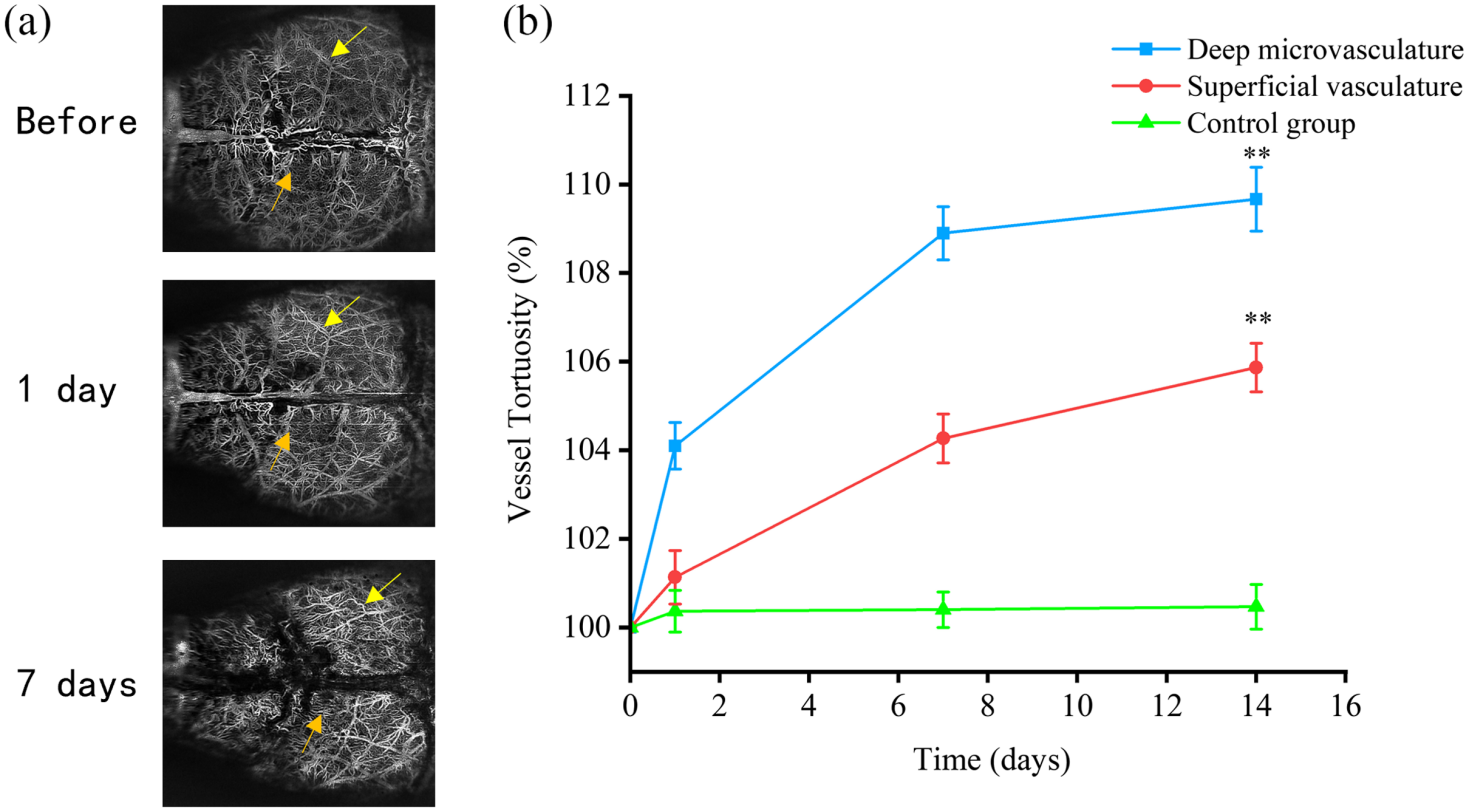
Cerebrovascular tortuosity before and after Aβ peptide injection. (a) Local OCT images indicate that the vessels became more tortuous on the 7th day (third row) compared with before treatment (first row). The vessels displayed tortuosity (yellow arrows) and twisting (orange arrows). Images in the same column are from the same location. Panel (b) shows the VT% change over time of the superficial vasculature (red lines), deep microvasculature (blue lines), and deep microvasculature in the control group (green lines). Each bar represents the group mean ± S.E.M, significant differences between certain groups, and before denoted **p<0.01.

Distinct vascular morphological changes, such as tortuous, twisted, and kinked structures, are often observed during AD pathological progression, both in postmortem examinations and in AD transgenic mouse models. In this study, we investigated the effects of Aβ on cerebral vascular morphology. Notably, some larger vessels became more twisted 7 days after Aβ injection [Fig. 7(a)]. We observed vascular tortuosity and deformation (yellow arrows), along with the formation of twisted and circular structures (orange arrows). The degree of vascular tortuosity was quantitatively assessed using VT, as shown in Fig. 7(b). The results indicated that post-injection, there was an increasing trend in vascular tortuosity. The VT of deep microvessels reached 108.9% and 109.7% (p<0.01) of their pre-injection values on days 7 and 14 post-injection, respectively [Fig. 7(b), blue line]. In comparison to the increasing trend observed in deep microvessels, the superficial vasculature showed a relatively subtle but significant change [Fig. 7(b), red line], reaching 104.3% and 105.9% (p<0.01) of its pre-injection values on days 7 and 14, respectively. However, there was almost no change in the VT of deep microvasculature in the control group [Fig. 7(b), green line].

**Fig. 8:**
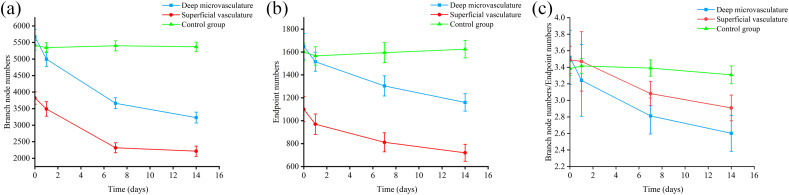
Impact of Aβ on vascular branching and rupture. (a) The number of branch nodes in superficial vasculature, deep microvessels, and control group based on different time points. (b) The number of endpoints in superficial vasculature, deep microvessels, and control group based on different time points. (c) The ratio of the number of branch nodes to the endpoint based on different time points.

In addition, we measured the number of branch nodes and endpoints, and the results showed a continuous decrease in the total number of branch nodes [Fig. 8(a)], which may suggest an anti-angiogenic effect. One week later, a significant reduction in the number of endpoints was observed [Fig. 8(b)], potentially indicating an increase in vascular fragmentation. Furthermore, the ratio of branch node numbers to endpoint numbers steadily declined over time [Fig. 8(c)], indicating that the decrease in branch nodes occurred at a faster rate than that of the endpoints, and was more pronounced in deep microvessels. However, there was almost no change in the control group.

## 4 Discussion and Conclusion

OCTA is a technique based on difference (typically amplitude difference) extraction between OCT images, whereas OCT utilizes backscatter light from the sample for imaging. Thus, OCTA is dependent on backscattering light change for flow signal detection. In OCT imaging, the light backscattering of deep tissue is inherently affected by upper tissues as deep tissue imaging requires a probing beam to penetrate the upper tissue. Hence, the component change in superficial vessels (blood cell flow) can cause a backscatter light change in deep tissue and introduce tail artifacts. On the other hand, irradiation fluctuation of the light source can also introduce backscatter light changes of all tissues between OCT B-scan. In this sense, it is also a tail artifact. The current tail artifact elimination methods focus on calculated difference signal (between B-scans) processing and are helpful in large vessel artifact elimination. However, the performance of small vessels and the elimination of light source fluctuation artifacts are not convincing. The proposed method utilizes intrinsic parameters of tissue OAC for difference extraction between OCT B-scans. Theoretically, the technique originally eliminates the influence of tail artifacts and improves the detection rate of small vessels in the deep cortex of the mouse.

Due to the similar embryonic origins, anatomical, and physiological characteristics of the retina and brain microvessels,^49^ the retina is considered a part of the central nervous system. It is connected to it through the optic nerve.^50^ Therefore, previous studies on AD using OCT mainly focused on the retina and choroid. For instance, Coppola et al.^51^ reported significant retinal nerve fiber layer thinning in AD patients using OCT. Katsimpris et al.^52^ further demonstrated that AD patients exhibit significantly reduced vessel density in the superficial vascular plexus (SVP) and parafoveal SVP using OCTA. The research on brain tissue in AD has primarily relied on post-mortem studies. For example, Lichtenegger et al.^53^ found differences in the characteristics of white matter, grey matter, and neuritic amyloid-beta plaques by performing a spectroscopic analysis of the optical coherence microscopy data. These previous studies have mainly focused on retinal blood vessels or *ex vivo* brain tissue research, lacking continuous *in vivo* monitoring of brain microvessels. Our analysis focuses on the *in vivo* continuous monitoring of the deep cortical vasculature, which may more directly reflect the pathological process of AD. In the future, we will build upon previous studies of retinal blood vessels to further explore the association between retinal and brain blood vessels in Alzheimer’s disease.

The insidious onset of AD means that patients frequently present with mild cognitive or memory deficits by the time they seek medical intervention. Consequently, there is a marked scarcity of samples in the preclinical stage, posing a significant barrier to early AD pathogenesis research. Against this backdrop, turning to robust animal models for foundational medical investigations becomes a prudent approach. Such models not only allow for a comprehensive exploration of the disease’s etiology and progression but also address the challenges posed by limited patient samples and the intricacies of longitudinal research. Utilizing AD mouse models, we harnessed SS-OCT technology to monitor their cerebrovascular systems longitudinally and undertook a detailed quantitative assessment of the vasculature.

Our results revealed an initial decline in the VPD induced by $A\beta_{1-42}$, dropping to 66% of pre-injection within 2 weeks. Several early-stage studies have highlighted a reduction in vascular density in AD mouse models and post-mortem AD patient brain specimens, with a pronounced impact on capillaries.^47^^,^^48^^,^^54^^–^^58^ Potential mechanisms for this vascular density decrease may include obstructions caused by the aggregation of antibodies and fibrillar proteins, leading to diminished perfusion.^48^^,^^59^ Moreover, an intensification of perfusion deficits, compounded by ensuing endothelial cell loss, might elucidate the vascular density reduction observed during this phase.^60^ Several reports have indicated either an increase or no change in vascular density,^61^^,^^62^ suggesting remodeling of the surviving vascular network in AD brains. This notion is supported by the denser vascular networks observed around Aβ deposits in young APP23 mice.^63^ The increased vascular density might be temporary, attributed to a transient release of vascular endothelial growth factor (VEGF).^48^^,^^58^^,^^64^ Conversely, a longitudinal study revealed that this transient increase was only observed in 14-month-old APP23 mice. At the same time, a significant decrease in vascular density was found at 20 months, ruling out the influence of aging.^48^ However, our findings align with observations in late-stage AD mice, where the decline in VPD might be due to reduced perfusion occurring at earlier time points and the subsequent loss of vascular cells during the later stages of monitoring. It is important to note that from a methodological standpoint, the VPD parameter in our study does not entirely correspond to the vascular density suggested in the aforementioned studies, particularly those involving the calculation of stained endothelial cells as a measure of vascular density *in vitro* post-mortem examinations.^54^ Instead, our OCT signal parameter accounts for blood perfusion and only includes functional vessels (RBC-perfused vessels). Nonetheless, our results suggest that soluble Aβ in cerebrospinal fluid can induce similar VPD trends in AD patients and animal models.^47^^,^^48^^,^^54^^–^^58^ Notably, the accentuated decline in the VPD of deep microvessels suggests that these regions are potential epicenters for early AD initiation and progression.

VL evaluates the length of blood vessels without considering vessel diameter. Over our 2-week observation period, VL exhibited a marked decrease, with more pronounced changes observed at the level of deep microvessels. VEGF, a potent stimulator of endothelial cell proliferation and angiogenesis, is found at elevated levels in the CSF of AD patients.^65^ When $A\beta_{1-42}$ enters the CSF, it competitively antagonizes the binding of VEGF-to-VEGF receptor-2 on endothelial cells,^66^ reducing the availability of VEGF in the vasculature.^67^^,^^68^ This not only suppresses the formation of new vessels but also, more crucially, induces regressive changes in existing vessels, leaving behind nonfunctional “string vessels” primarily composed of connective tissue and lacking endothelial cells.^69^ This process might be relevant to the pathophysiology of AD and could explain the significant reduction in VL observed during our 2-week monitoring period, although further studies are needed to confirm the presence of string vessels. We found that CSF-$A\beta_{1-42}$ promoted a reduction in VL, particularly in microvessels, aligning with previous observations in AD patients and mouse models that demonstrate Aβ-dependent vascular degeneration.^55^^,^^70^^–^^72^ Our research also suggests that, compared with superficial vasculature, VL and VPD in deep microvessels are equally more sensitive to $A\beta_{1-42}$, indicating their potential practicality as indicators for early AD diagnosis.

VAD represents the mean diameter across the entire vasculature, derived through distance transformation of binarized OCT angiography images and calculated from the vessel skeleton (the central axis of vessels) based on OCT signal intensity. In our study, a moderate yet significant increase in microvascular VAD (∼105.2%) was observed 7 days following intracerebroventricular administration of $A\beta_{1-42}$, suggesting that cerebrospinal fluid amyloid-beta has a mild vasodilatory effect. In AD mice, reduced perfusion and hypoxia could induce vasoconstriction through the upregulation of the vasoconstrictor endothelin-1.^73^ Furthermore, Aβ itself acts as an activator of endothelin-1, exhibiting direct vasoconstrictive effects.^74^ This corresponds with the slight decrease in VAD observed on day 14 post-injection. Concurrently, studies monitoring transgenic AD mice (APP23) over 20 months have reported an average vascular diameter and size index increase despite a slight constriction observed at 14 months.^48^ Our findings are more consistent with the pathological characteristics observed in the later stages of AD. More importantly, our research results also indicate that the vasodilatory effect of $A\beta_{1-42}$ may be predominantly observed in deep microvessels.

Vascular deformations, such as tortuous, twisted, and looped vessels [Fig. 7(a)], were observed in the later stage of our monitor. These vascular morphological alterations were also seen in many patients with advanced AD.^54^^,^^60^^,^^75^^,^^76^ By computation of the ratio between the actual distance and the linear distance across each branching terminus, the labeled skeletal OCT images enabled us to quantify these vascular deformations. These vascular morphological changes have also been observed in many late-stage AD patients. By calculating the ratio of the actual distance between each branch terminus and the straight-line distance, the labeled skeletonized OCT images allow us to quantify these vascular deformation morphologies. The results indicate a significant increase in the curvature of deep microvessels, with a mild elevation in the curvature of superficial vasculature. The findings suggest that changes in vascular morphology predominantly occur at the level of deep microvessels. Such structural alterations could substantially affect the local circulation as these abnormal configurations may elevate vascular resistance, thus perturbing the hemodynamic balance within the local vascular network.^54^^,^^77^ The increased density of “string vessels” due to endothelial cell degeneration may be related to reduced local perfusion and metabolic dysfunction.^60^ Dysfunctional vessels may also stem from abnormal angiogenesis, partly attributable to Aβ’s antagonistic effect on VEGF.^66^ This observation is consistent with our results as we noted a significant reduction in the number of branch nodes at 14 days post-injection [Fig. 8(a)], indicating that Aβ in the lymphatic system exhibits properties that inhibit angiogenesis and branching, consistent with previous studies.^78^ $A\beta_{1-42}$ hampers angiogenesis by inhibiting VEGF’s expression, secretion, and function.^66^^,^^79^^,^^80^ Supplementing VEGF in AD transgenic mice has shown cognitive improvement, enhanced angiogenesis, and reduced amyloid burden.^81^^,^^82^ Evidence also suggests that Aβ can enhance angiogenesis, yet these effects are likely to be localized and inefficient. The resulting neovascularization may be of low quality and susceptible to early degeneration, attributed to vascular cell death and the decreased expression of growth factors.^83^^,^^84^ We also measured the number of branching points and endpoints, which significantly decreased following the injection of Aβ, suggesting an increase in vascular fragments or a rise in acellular and collapsed microvessels—a microvascular morphology observed in the late stages of AD.^85^ The ratio of branching nodes to endpoints steadily declined over time, possibly indicating that the once-rich vascular network was becoming sparser and more dispersed. The quantification of branching and endpoint numbers is crucial for estimating reduced perfusion and regional hypometabolism.

In summary, this study initially addressed the analysis of the tail effect. To tackle the origins of this effect, we introduced the OAC decorrelation algorithm. We notably suppressed the tail effect and enhanced image quality by contrasting the vascular images derived from OCT signals and OAC values. Using SS-OCT, we monitored the morphological changes of deep microvessels in a live AD mouse model. Research has found that after the injection of $A\beta_{1-42}$, we observed a significant and sustained decrease in the VPD and VL of deep microvessels. In addition, a slight but significant increase in VT and VAD was observed 14 days post-injection. These findings align with vascular pathological alterations frequently seen in advanced AD. These findings align with the vascular pathological alterations often observed in severe Alzheimer’s disease, suggesting that an excessive accumulation of $A\beta_{1-42}$ in the glymphatic system can cause significant harm, particularly affecting deeper microvessels. Compared with superficial vasculature changes, the alterations in deep microvessels were more sensitive, suggesting that deep microvessels could potentially be an early pathological hallmark of AD. This offers a novel direction and approach for early AD detection and drug development in the future. In addition, our research highlights using OCT as a viable tool in drug discovery for tracking changes in brain vasculature and tissue during therapeutic interventions.

## Data Availability

The data used in this study can be obtained from the first or corresponding author, provided the request is justified and aligns with the policies of the institution.
